# Cancer-Associated Fibroblasts Facilitate Squamous Cell Carcinoma Lung Metastasis in Mice by Providing TGFβ-Mediated Cancer Stem Cell Niche

**DOI:** 10.3389/fcell.2021.668164

**Published:** 2021-08-30

**Authors:** Xueke Shi, Jingjing Luo, Kelsey J. Weigel, Spencer C. Hall, Danfeng Du, Fanglong Wu, Michael C. Rudolph, Hongmei Zhou, Christian D. Young, Xiao-Jing Wang

**Affiliations:** ^1^State Key Laboratory of Oral Diseases, National Clinical Research Center for Oral Diseases, West China Hospital of Stomatology, Sichuan University, Chengdu, China; ^2^Department of Pathology, University of Colorado Anschutz Medical Campus, Aurora, CO, United States; ^3^Division of Endocrinology, Metabolism and Diabetes, University of Colorado Anschutz Medical Campus, Aurora, CO, United States; ^4^Veterans Affairs Medical Center, VA Eastern Colorado Health Care System, Aurora, CO, United States

**Keywords:** squamous cell carcinoma, transforming growth factor-beta, cancer associated fibroblast, lung metastasis, cancer stem cell

## Abstract

Cancer-associated fibroblasts (CAFs) have been shown to enhance squamous cell carcinoma (SCC) growth, but it is unclear whether they promote SCC lung metastasis. We generated CAFs from *K15.KrasG12D.Smad4^–/–^* mouse SCCs. RNA expression analyses demonstrated that CAFs had enriched transforming growth factor-beta (TGFβ) signaling compared to normal tissue-associated fibroblasts (NAFs), therefore we assessed how TGFβ-enriched CAFs impact SCC metastasis. We co-injected SCC cells with CAFs to the skin, tail vein, or the lung to mimic sequential steps of lung metastasis. CAFs increased SCC volume only in lung co-transplantations, characterized with increased proliferation and angiogenesis and decreased apoptosis compared to NAF co-transplanted SCCs. These CAF effects were attenuated by a clinically relevant TGFβ receptor inhibitor, suggesting that CAFs facilitated TGFβ-dependent SCC cell seeding and survival in the lung. CAFs also increased tumor volume when co-transplanted to the lung with limiting numbers of SCC cancer stem cells (CSCs). *In vitro*, CSC sphere formation and invasion were increased either with co-cultured CAFs or with CAF conditioned media (which contains the highest TGFβ1 concentration) and these CAF effects were blocked by TGFβ inhibition. Further, TGFβ activation was higher in primary human oral SCCs with lung metastasis than SCCs without lung metastasis. Similarly, TGFβ activation was detected in the lungs of mice with micrometastasis. Our data suggest that TGFβ-enriched CAFs play a causal role in CSC seeding and expansion in the lung during SCC metastasis, providing a prognostic marker and therapeutic target for SCC lung metastasis.

## Introduction

Squamous cell carcinomas (SCCs) arise from stratified epithelia, and the most relevant organ sites are the skin and oral cavity where high UV irradiation, tobacco carcinogens, or human papillomavirus (HPV) infection are initiating events. The worst outcome of SCC is death caused by distant metastasis, most commonly to the lung (Bohnenberger et al., 2018; Alfieri et al., 2020). Lung metastasis is the process of cancer cells disseminating from a primary SCC, entering into blood vessels or lymphatic vessels (intravasation), survival and traveling, and moving out of vessels (extravasation) into the lung, and survival and expansion in the lung thereafter (Chaffer and Weinberg, 2011).

The major challenge for studying mechanisms of SCC lung metastasis is the shortage of spontaneous SCC lung metastasis models mimicking the entire metastatic process. We previously generated an aggressive SCC mouse model driven by *Kras^*G*12*D*^* mutation and *Smad4* deletion (*Smad4^–/–^*) in keratin 15 (K15)-positive stem cells of stratified epithelial tissues, i.e., hair follicle bulge or tongue papillae (White et al., 2013). *K15.Kras^*G*12*D*^.Smad4^–/–^* mice develop spontaneous SCCs, some of which metastasized to the lung (White et al., 2013). In that study, we identified that cancer stem cells (CSCs) derived from mutant stem cells in these SCCs have a higher invasive ability than non-CSCs (White et al., 2013). Taken together, CSCs are expected to have a higher chance than non-CSCs to invade from the primary site, survive through trafficking and engraft at the metastatic site. CSCs have self-renewal ability and the capacity to generate the progeny cells that constitute the tumor and are resistant to cell death (Chaffer and Weinberg, 2011; Malanchi et al., 2011). However, how CSCs as SCC metastasis “seeds” interact with their “soil” during metastasis, remains to be assessed.

Cancer stem cells depend upon the stromal niche to maintain their stem-like properties (Malanchi et al., 2011; Plaks et al., 2015). Among the most abundant cells in the stromal niche, cancer-associated fibroblasts (CAFs) communicate with cancer cells *via* cell–cell contacts and production of chemokines, cytokines, and factors that contribute to SCC progression (Markwell and Weed, 2015; Hogervorst et al., 2018; Peltanova et al., 2019). CAFs have been shown to enhance SCC cell proliferation, migration, and invasion *in vitro* (Li et al., 2015; Hogervorst et al., 2018). In an *in vivo* model, CAFs from skin SCC possess a proinflammatory gene signature that promotes tumor growth (Erez et al., 2010). In addition, we have previously shown that CAFs facilitate oral SCC (OSCC) tumor growth *in vivo* (Meng et al., 2014). Data from *in vitro* experiments have shown that CAFs enhance the self-renewal of CSCs in different cancers including HNSCCs (Chen et al., 2014; Álvarez-Teijeiro et al., 2018; Su et al., 2018; Le et al., 2019). However, it remains to be determined whether CAFs promote CSC invasion and whether enhancement of CSC properties (self-renewal and invasion) is sufficient to impact SCC metastasis *in vivo*, and if so, at what stage of metastasis and *via* what mechanisms.

In the current study, we transplanted metastatic SCC cells derived from *K15.Kras^*G*12*D*^.Smad4^–/–^* mice and CAFs derived from the stroma of these SCCs into C57BL/6J or athymic mice. Using SCC-CAF co-transplantation, we sought to determine: whether CAFs enhance SCC lung metastasis; and if so, at which stage of lung metastasis; whether CAFs promote CSC self-renewal and invasion resulting in more lung metastasis; and, what signaling pathways drive CAF-influenced SCC lung metastasis. Our study revealed that CAF’s primarily influence the distant metastatic site for CSCs to be seeded and expanded in the lung in a transforming growth factor-beta (TGFβ)-dependent manner. Use of a clinically relevant TGFβ inhibitor to inhibit CAF-promoted lung engraftment provides preclinical evidence of this critically important event and suggests that TGFβ from CAFs is a major contributor to metastasis. And, therapeutic targeting of SCC metastasis with TGFβ inhibitors is feasible and worthy of further research.

## Materials and Methods

### Establishment of Cell Lines and Cell Culture

#### SP Flow Cytometry Sorting and Culture

Mouse SCC cell lines A223, B931 are derived from *K15.Kras^*G*12*D*^.Smad4^–/–^* C57BL/6J mice as previously described (White et al., 2013). All cell lines were cultured in Dulbecco’s modified Eagle medium (DMEM) containing 10% fetal bovine serum (FBS). Efflux of Hoechst 33342 dye to isolate the Hoechst-negative SP cells, a subpopulation of metastasis associated CSCs (White et al., 2013), was performed at the University of Colorado Cancer Center Flow Cytometry Shared Resource as previously described (White et al., 2013).

#### CAFs/Normal Tissue-Associated Fibroblasts Isolation, Culture, Purification

Cancer-associated fibroblasts were isolated from transplanted tongue SCC tumors and normal tissue-associated fibroblast (NAFs) were isolated from normal tongues of independent mice using enzymatic digestion as described in Supplementary Materials and Methods (Mazzocca et al., 2010; Yang et al., 2016). Two independent CAF cell lines and two independent NAF cell lines (from four different mice) were established and cultured in DMEM containing 10% FBS.

### RNA-seq and Analysis

Total RNAs were extracted using RNeasy Plus Mini Kit (Qiagen, Germantown, MD, United States). Total RNA (100 ng) was used as input to construct mRNA libraries using the NuGEN Universal Plus mRNA-Seq protocol part no. 9133 (NuGEN, Redwood City, CA, United States). Sequencing was done on an Illumina NovaSEQ 6000 instrument using an S4 flow cell and 2 × 150 paired end sequencing (Illumina, San Diego, CA, United States). A custom computational pipeline consisting of the open-source gSNAP, Cufflinks, and R was used for alignment and discovery of differential gene expression (Presby et al., 2019). Briefly, each high-resolution sequencing read generated by each sample was mapped to the mouse genome (GMAPDBv2) using gSNAP, Cufflinks calculated the prevalence of transcripts from each known gene, and each gene was expressed as transcript levels in fragments per kilobase of exon per million mapped reads (FPKM). From this, significant differentially expressed mRNA profiles were identified using ANOVA in R with an FDR of *P* < 0.05. These expression data were evaluated by GSEA against the Hallmark Gene Sets and Canonical Pathways KEGG Gene Sets using GSEA 4.0.3 software downloaded from gsea-msigdb.org. Raw sequencing files are available in the Sequence Read Archive (SRA^1^). Accession SRR13996315 and SRR13996316.

### Tumor Transplantation and Treatment

Animal studies were reviewed and approved by the Institutional Animal Care and Use Committee of the University of Colorado Anschutz Medical Campus. C57BL/6J mice (Jackson Laboratory) or athymic nude (Charles River Laboratories) at 8- to 10-week of age were used as tumor and fibroblast transplantation recipients. A223 SCC cells were transplanted with or without fibroblasts to female C57BL/6J mice. SCC cells or their sorted CSCs from B931 were transplanted into athymic nude mice because they are incapable of tumor formation in immunocompetent C57BL/6J mice. A total of 1,000 total B931 were transplanted subcutaneously to the right flank of anesthetized mice. For SCC cell-fibroblasts tail vein co-injection and SCC cell-fibroblast subcutaneous co-transplantation, see Supplementary Materials and Methods. For SCC or CSC (SP) co-transplantation with fibroblasts to the lung, a total of 1,000 SCC cells or 100 SP cells with or without 5,000 CAFs/NAFs was injected into the mouse left lung (unless otherwise indicated). One lung tumor was initiated on the left side after cells were transplanted to the left lung, volume was calculated using the following formula: Volume (mm^3^) = (length × width × depth)/2. Metastasis to the right lung was assessed by counting the number of metastases in H&E stained sections of the right lung. Complete methods are described in the Supplementary Materials and Methods.

For TGFβ inhibitor treatment, mice were treated with TGFβ inhibitor (LY2109761 or LY2157299, 150 mg/kg/day) by oral gavage or an equal volume of vehicle (Tran et al., 2017) (1% carboxymethylcellulose, 0.5% sodium lauryl sulfate, 0.085% povidone, and 0.05% antifoam) daily for 19–25 days before being sacrificed and lungs harvested. LY2109761 was used in early experiments and we later switched to the clinical drug version LY2157299 (galunisertib) to assure translational relevance. For more information, see Supplementary Materials and Methods.

### Histology

Primary tumors and lungs harvested at the endpoint of the study were embedded in paraffin, sectioned, and stained with Hematoxylin and eosin staining (H&E). Histopathology of primary tumors, lung tissue, micrometastasis, and metastasis were evaluated on H&E sections.

### Conditioned Media Collection

#### CAF/NAF CM Collection for CSC Sphere Formation

Cancer-associated fibroblasts/NAFs seeded at the same density (about 90%) were incubated in serum-free media and conditioned media (CM) were harvested after 36 h. CM were collected, centrifuged to remove cellular debris, and used immediately for CSC sphere formation assays (described below).

#### CM Collection and TGFβ1 ELISA

1 × 10^4^ CSCs or SCCs with or without 5 × 10^4^ CAFs or NAFs were cultured in the CSC media (serum-free media) for 36 h. CM were collected, centrifuged to remove the cells and debris, and used for TGFβ1 ELISA (R&D Systems, Minneapolis, MN, United States) following the manufacturer’s instructions as reported previously (Li et al., 2004). The optical density (OD) of each well was detected using a microplate reader set to 450 nm. Tumor lysates were normalized to the same protein concentration prior to TGFβ1 ELISA.

### CSC Sphere Assay

Squamous cell carcinoma cells were transduced with green fluorescence protein (GFP)-expressing lentivirus and selected by flow cytometry sorting as previously described (White et al., 2013). CAFs or NAFs were transduced with NucLight Red Lentivirus (Essen Biosciences, Ann Arbor, MI, United States) followed by selection with 2.5 μg/mL bleomycin to obtain cells stably expressing nuclear red fluorescence protein (RFP) (RFP^+^ CAF/NAF). Cells were plated in ultra-low attachment (ULA) plates (Corning) to assess sphere-forming capacity. For direct co-culture, 100 GFP^+^ CSCs with or without 500 RFP^+^ CAFs/NAFs were seeded in each well of a 24-well ULA plate. To assess whether CM of CAFs/NAFs induced sphere formation, 100 μL CM of CAFs/NAFs were mixed with 50 GFP^+^ CSCs (in 50 μL CSC media) in each well of a 96-well ULA plate. TGFβ inhibitor (LY2157299) was applied at a final concentration of 5 μmol/L or an equal volume of DMSO was added as a negative control. After culturing for 7–10 days, whole well imaging was performed using an IncuCyte Zoom live cell imaging instrument at the University of Colorado Cell Technologies Shared Resource. Spheres with diameter >100 mm were counted.

### Invasion Assay

Invasion assay was performed as previously described (White et al., 2013). Transwell Matrigel-coated invasion chambers (BD Biosciences, 8 μm pore membranes) were prepared according to the manufacturer’s instructions. A total of 50,000 RFP^+^ CAFs/NAFs were seeded in the bottom well 24 h before 10,000 GFP^+^ CSCs were added in the top chamber. TGFβ inhibitor (LY2109761) was applied at final concentration of 5 μmol/L or an equal volume of DMSO was added as a negative control. After 48 h, uninvaded cells were removed from the upper chamber with a moist cotton swab and invaded cells below the top chamber were fixed in 10% formalin and stained with hematoxylin. Three fields at 100× magnification were captured and counted in each of three replicates.

### RT-qPCR

Unconditioned media and CAF CM were prepared as described above and applied to 100,000 recipient A223 cells in sphere culture and incubated 24 h. RNA was harvested as described above. RT-qPCR was performed using 40 ng RNA, Brilliant II QRT-PCR 1-Step Master Mix (Agilent, Santa Clara, CA, United States) and TaqMan gene expression assays for *Junb* (Mm04243546_s1), *Spp1* (Mm00436767_m1), *Vegfa* (Mm00437306_m1), and *Gapdh* (Mm99999915_g1) (ThermoFisher, Rockford, IL, United States). The expression of each gene relative to *Gapdh* was determined using the 2^ΔCT^ method and normalized to the unconditioned media control.

### IHC Staining

#### IHC of Mouse Tissues

IHC staining was performed as previously described (Lu et al., 2006; Luo et al., 2019). The primary antibodies used for mouse tissues were α-smooth muscle actin (αSMA, 1:500, CST), p-SMAD3 (1:250, Abcam, Cambridge, MA, United States), cleaved-caspase 3 (1:200, CST), CD31 (1:200, CST), Ki67 (1:400, Abcam), and TGFβ1 (1:300, Abcam). All sections that contained tumor tissue in each group were stained and quantified. For p-SMAD3, cleaved-caspase 3, CD31, and Ki67, five 200× fields/lung tumor sample were captured, and positive staining cell number or positive staining area for each field was quantified by Image-Pro Plus 6.0 (Media Cybernetics, Rockville, MD, United States). The positive staining fraction (%) or positive staining cell number value of each field/sample was averaged to obtain the positive staining fraction or cell number for each sample.

#### IHC of Human OSCC Samples

West China Hospital of Stomatology, Sichuan University, China approved the experiment as being human subject exempted. De-identified human tissue paraffin sections were used in this study. All tissues were from primary OSCC tumor biopsies from patients without prior cancer therapy. A total of 6–7 non-lung metastatic OSCCs and 7–10 lung metastatic OSCCs were used for IHC staining using primary antibodies against TGFβ1 (1:100, Abcam), p-SMAD2 (1:100, Invitrogen), or p-SMAD3 (1:100, Abcam). A total of 5–10 fields at 200× magnification were captured per OSCC sample. Quantification of area stained and the integrated optical density (IOD) of indicated markers in each image were measured by Image-Pro Plus 6.0 (Media Cybernetics). Average optical density (Hu et al., 2014) (AOD = IOD/Area) was used in this study for statistical analysis. The mean AOD of 5–10 fields was the AOD value for one OSCC sample.

### Statistical Analyses

Statistical analyses for comparisons between two groups were performed using SPSS version 24.0 for Windows (IBM, New York, NY, United States). Normality test for group data sets was determined by Shapiro–Wilk normality test. Statistical differences between two groups were performed by unpaired parametric Student’s *t*-test or non-parametric Mann–Whitney exact test, as appropriate. Statistical differences between more than two groups were determined by one way ANOVA with Tukey’s multiple comparison test using GraphPad Prism version 9 (GraphPad Software, San Diego, CA, United States). Individual data points represented values of technical or biological replicates, and they were shown as mean ± SEM. ^ns^*P* > 0.05, ^∗^*P* < 0.05, ^∗∗^*P* < 0.01, ^∗∗∗^*P* < 0.001.

## Results

### TGFβ Signaling Is Enriched in Mouse SCC CAFs

We transplanted SCC cells from A223 line into the tongues of female C57BL/6J recipients. Once the tumor was developed, we established CAF cell lines as previously reported (Yang et al., 2016). We confirmed that CAF lines are free of tumor cells that would harbor *Smad4* deletion, express *Cre* transcript, and KRAS^G12D^ protein (Supplementary Figures 1A,B). We also established normal fibroblast cell lines from the tongue of female non-tumor bearing mice. CAFs and NAFs did not express keratins (Supplementary Figures 1B,C), which are still expressed in poorly differentiated SCC cells, indicating fibroblast cell lines are not contaminated with epithelial cells. The fibroblasts expressed fibroblast activation protein (FAP) and vimentin (Supplementary Figure 1C) demonstrating their fibroblast phenotype.

**FIGURE 1:**
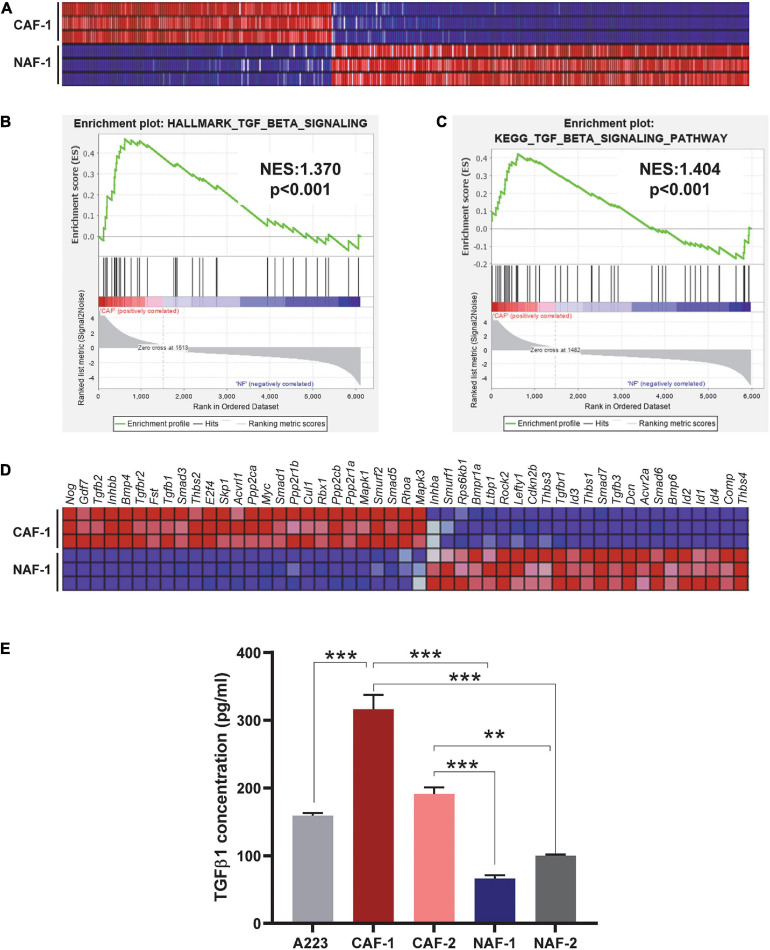
TGFβ signaling is activated in CAFs. **(A)** Heat map of all differentially expressed genes comparing three technical replicates of the CAF-1 to NAF-1 cell lines (*P* < 0.05). **(B,C)** GSEA analysis of the Hallmark Gene Sets **(B)** and the KEGG Pathways Gene Sets **(C)** identified enrichment of TGFβ signaling in CAFs compared to NAFs. NES: normalized enrichment score. **(D)** Heat map of differentially expressed genes between CAFs and NAFs in the KEGG TGFβ signaling pathway. **(E)** Detection of TGFβ1 in conditioned media of SCCs, CAFs, and NAFs was performed using ELISA. Either three or four technical replicates were conducted for each cell type and two independent CAF cell lines and two independent NAF cell lines were utilized. CAF-1 and CAF-2 cell lines were derived from two different, independent SCC tumors. NAF-1 and NAF-2 cell lines were derived from the tongues of two different wild type non-tumor-bearing C57BL/6J mice. **P < 0.01, ***P < 0.001.

To understand the molecular landscapes of CAFs vs. NAFs, we performed mRNAseq with three technical replicates of one CAF cell line and one NAF cell line. Overall, CAFs were significantly different from NAFs with 2007 significantly differentially expressed genes identified between the two groups (*P* < 0.05) (Figure 1A). GSEA interrogation against the Hallmark Gene Sets and the KEGG Pathway Gene Sets was performed and both analyses identified enrichment of TGFβ signaling (Figures 1B,C). *Tgfb1*, *Tgfb2*, and TGFβ signaling mediators were highly expressed in CAFs whereas *Tgfb3* and inhibitory *Smads* (*Smad6* and *Smad7*) were reduced in CAFs (Figure 1D). Because TGFβ1 is the predominant TGFβ ligand in tumors (Martin et al., 2020), we further examined if CAFs are a major source of TGFβ1 protein. TGFβ1 ELISA analysis showed that CM from CAFs derived from A223 SCCs produced more TGFβ1 protein than cultured A223 cells, whereas CM from NAFs produced much less TGFβ1 protein (Figure 1E).

### CAFs Increase SCC Engraftment and Expansion in the Lung in a TGFβ-Dependent Manner

**FIGURE 2:**
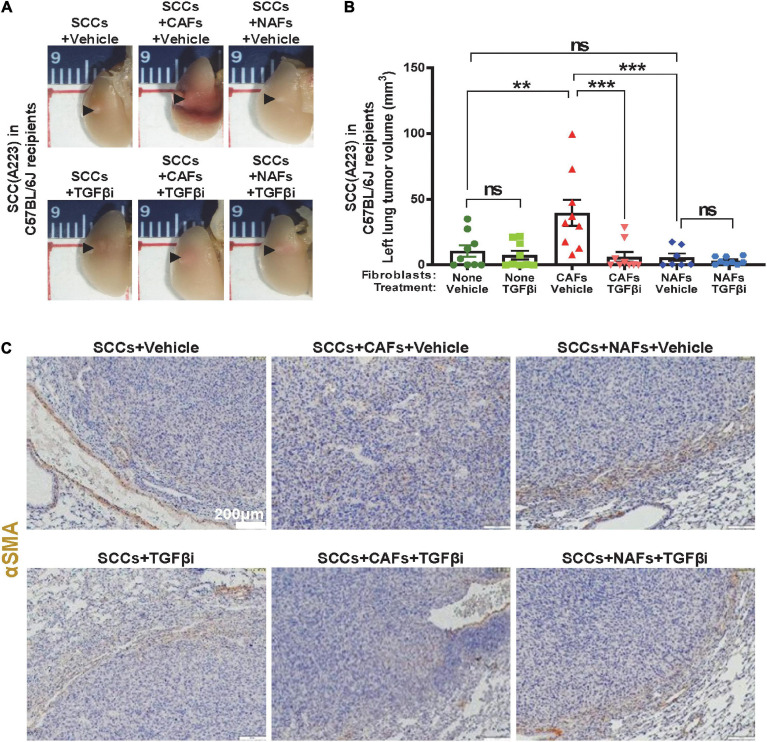
Cancer-associated fibroblasts contributed to TGFβ-dependent SCC seeding to the lung in immunocompetent recipients. **(A)** 1,000 A223 SCC cells and 5,000 of the indicated fibroblast types were co-transplanted to left lung, mice were treated daily with vehicle or TGFβ inhibitor (“TGFβi”) LY2109761 and lungs were harvested on day 21 and imaged under 1× magnification using a dissecting microscope. Representative gross images are presented. **(B)** Quantification of lung-seeded tumor volume (from gross samples) at the end point, ^*ns*^*P* > 0.05, **P < 0.01, ***P < 0.001. **(C)** Representative αSMA IHC stained images.

We assessed if TGFβ activation in CAFs is sufficient to affect metastasis *in vivo*. We first co-transplanted 1,000 SCC cells (A223 or B931) with 5,000 CAFs, 5,000 NAFs or without fibroblasts to the flank skin, tail vein, or directly to the left lung of the recipient C57BL/6J or athymic nude mice. These routes of transplant mimic different stages of metastasis (intravasation, survival, extravasation, seeding, and expansion in the lung), respectively. CAFs, but not NAFs, increased SCC expansion in the lung of C57BL/6J recipients upon co-injection directly to the left lobe of the lung, and TGFβ inhibitor LY2109761 attenuated the effect of CAFs on SCC expansion in the lung (Figures 2A,B). CAFs had no effect on primary tumor growth, or lung metastasis when co-injected into the skin (from the primary site) or through tail vain injection (intravenous trafficking) (Supplementary Figures 2A–C). Additionally, CAFs only affected tumor volumes and lesions in the left lung but not metastasis to the right lung (Supplementary Figure 3), indicating that CAFs promoted SCC cell seeding and expansion but not trafficking in vessels or invasion within the lung. IHC staining of αSMA, a commonly used marker for activated fibroblasts (Sridhara et al., 2013; Luksic et al., 2015; Kalluri, 2016; Maqsood et al., 2020), was used to determine the position of activated fibroblasts or CAFs. αSMA positive fibroblasts were distributed among tumor cells when CAFs were co-injected. However, αSMA positive fibroblasts were mainly located in the periphery of the tumor in other groups (Figure 2C). As αSMA also stains vessels, it is critical to note only the staining independent of vessels.

**FIGURE 3:**
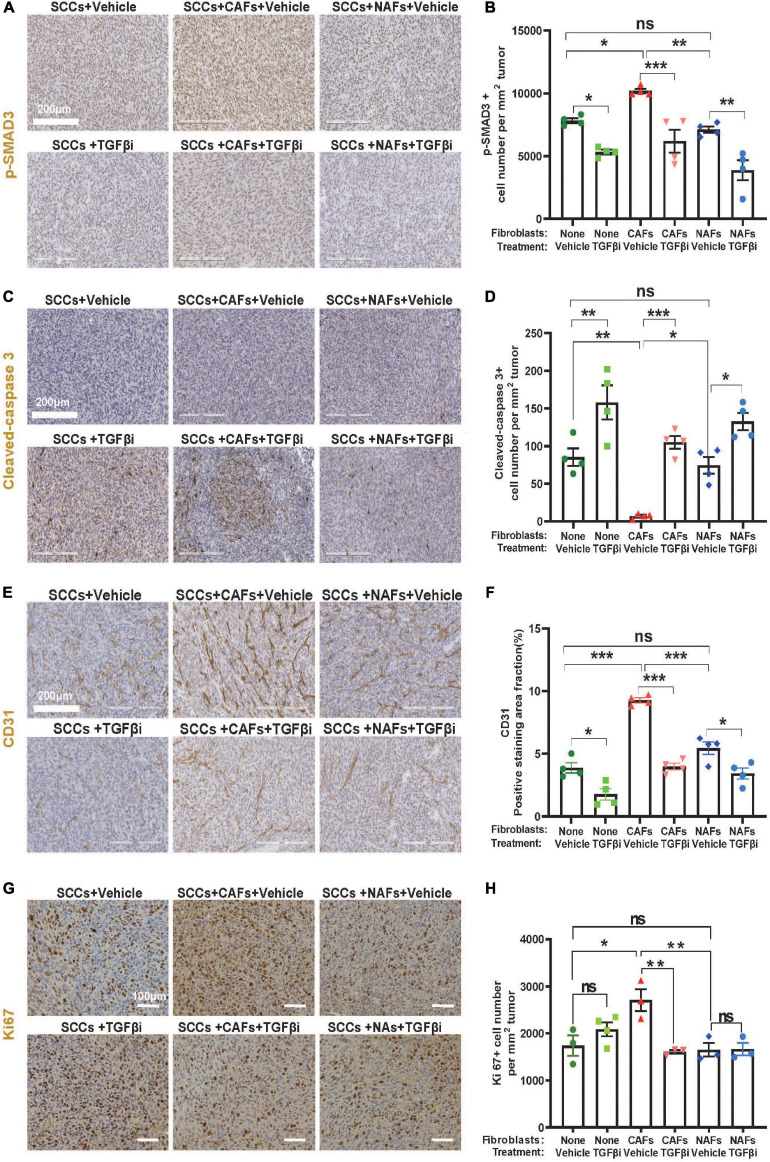
Increased proliferation and angiogenesis and decreased apoptosis in CAF-co-transplanted, lung-seeded tumors is attenuated by TGFβ inhibition. **(A,B)** Representative p-SMAD3 IHC stained images and quantification in lung-seeded A223 tumors. Scale bar: 200 μm. **(C,D)** Representative cleaved-caspase 3 IHC stained images and quantification in lung-seeded A223 tumors. Scale bar: 200 μm. **(E,F)** Representative CD31 IHC stained images and quantification in lung-seeded A223 tumors. Scale bar: 200 μm. **(G,H)** Representative Ki67 IHC stained images and quantification in lung-seeded A223 tumors. Scale bar: 100 μm. ^*ns*^*P* > 0.05, *P < 0.05, **P < 0.01, ***P < 0.001.

Compared to lung tumors derived from SCC + NAF co-transplantations, lung tumors derived from co-transplanted SCC + CAFs showed increased p-SMAD3, a surrogate marker of TGFβ activation (Figures 3A,B), decreased cleaved-caspase 3, a marker of apoptosis (Figures 3C,D), elevated CD31, a marker of endothelial cells (Figures 3E,F) and elevated Ki67 (Figures 3G,H) in vehicle-treated mice. Further, TGFβ inhibitor LY2109761 attenuated p-SMAD3 and CD31, and increased cleaved-caspase 3 in all groups (Figures 3B,D,F), validating on-target activity of the inhibitor and suggesting that apoptosis suppression and increased angiogenesis require TGFβ signaling and a positive relationship between CD31-marked angiogenesis and apoptosis suppression. TGFβ inhibitor only attenuated Ki67 in tumors derived from SCC + CAF co-injection (Figures 3G,H), suggesting that elevated TGFβ in CAFs contributes to SCC growth after seeding to the lung, but SCC proliferation is not driven by intrinsic SCC TGFβ signaling.

### CAFs Increase CSC Seeding to the Lung in a TGFβ-Dependent Manner

**FIGURE 4:**
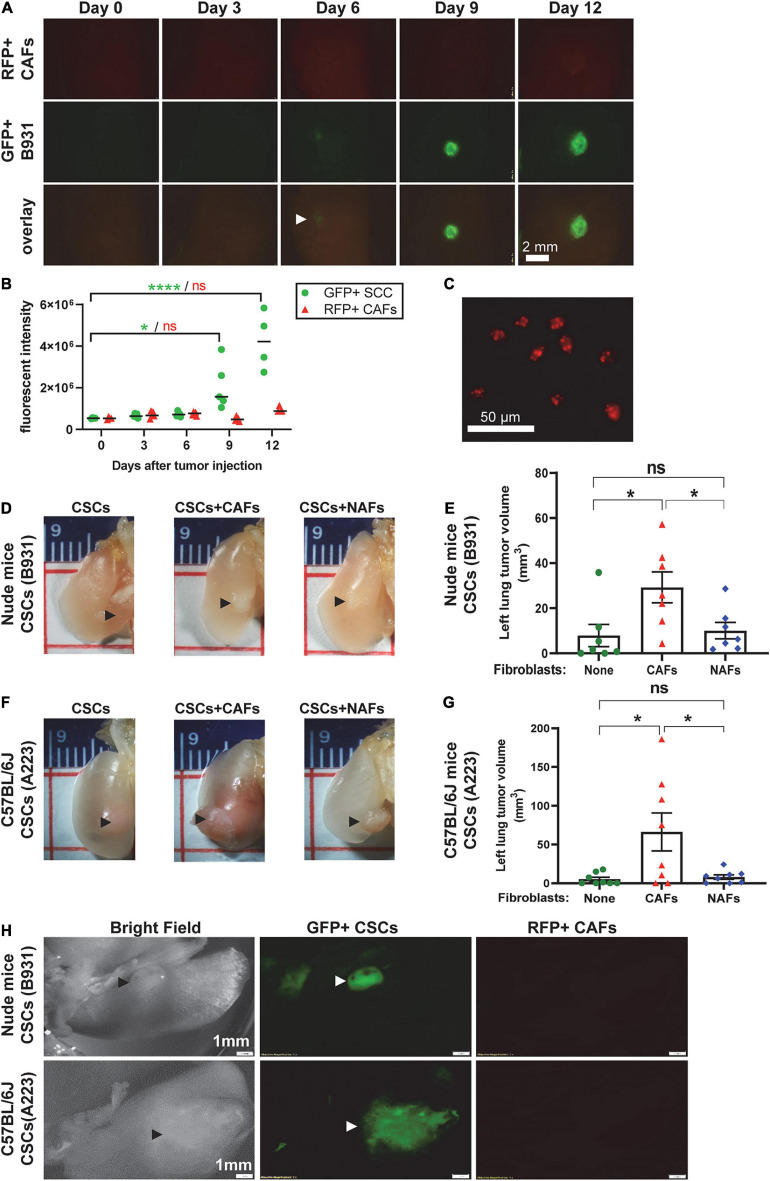
Cancer-associated fibroblasts did not expand with CSC cells but facilitated CSC cell seeding and expansion in the left lung. Transplanted A223 and B931 CSCs were used in all experiments as indicated. **(A,B)** 10,000 GFP^+^ B931 cells were co-transplanted with 50,000 RFP^+^ CAFs directly to the left lung and 4–5 mice were sacrificed on days 0, 3, 6, 9, and 12 post-transplant and left lungs imaged as described in panel **(F)** to detect GFP and RFP positive lesions. Representative images of lungs at each time point are presented in panel **(A)** and quantification of GFP and RFP intensity is presented in panel **(B)**. **(C)** RFP^+^ CAFs were imaged with an inverted fluorescent microscope using a 10× objective to demonstrate RFP positivity. **(D–G)** Representative images of left lung tumor, and quantification of left lung tumor volume in nude mice **(D,E)** and C57BL/6J mice **(F,G)** after 100 flow sorted side population (CSCs) of the indicated SCC cell lines were directly injected to the left lung with and without 5,000 CAFs or NAFs co-transplanted. Lungs were harvested and imaged from nude mice on day 19 and from C57BL/6J mice on day 25. **(H)** Brightfield and fluorescent imaging of gross tumors in the left lung tumor immediately after harvest was performed using a fluorescent dissecting microscope. SCCs were labeled with green fluorescence protein (GFP) and CAFs with red fluorescence protein (RFP). Only SCCs but not CAFs were detected. Scale bar: 1 mm. ^*ns*^*P* > 0.05, *P < 0.05, ****P < 0.0001.

To determine whether CAFs grow and expand with transplanted tumor cells, we labeled SCC cells with GFP and CAFs with RFP. After co-transplantation of 1,000 GFP^+^ SCC cells with 5,000 RFP^+^ CAFs directly to the left lung, we were unable to detect RFP^+^ CAFs (data not shown). We therefore increased the cell transplant numbers 10-fold and monitored relative levels of RFP^+^ CAFs and GFP^+^ SCC cells at multiple time points post transplantation using fluorescent dissecting microscopy. Expansion of GFP^+^ SCC cells was apparent as early as 6 days post-transplant and the intensity of the lesions continued to increase over time (Figures 4A,B). In contrast, the levels of RFP^+^ CAFs were maintained at the same intensity level for the entire assay (Figures 4A,B), suggesting that transplanted CAFs do not appreciably expand with the tumor cells at this level of detection. To be clear, RFP^+^ CAFs could be detected by traditional fluorescent microscopy (Figure 4C).

**FIGURE 5:**
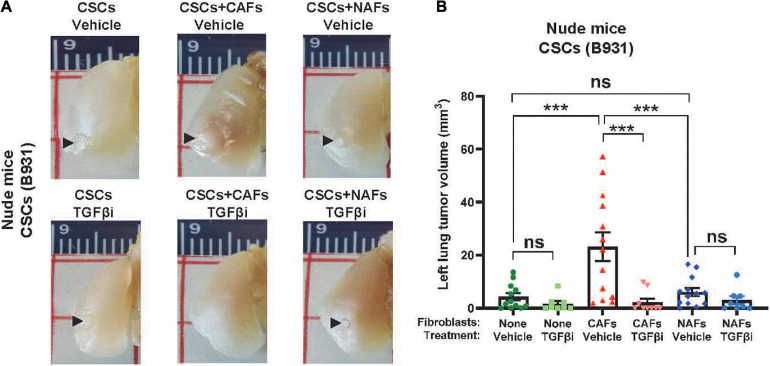
Cancer-associated fibroblasts facilitated TGFβ-dependent CSC cell seeding and expansion in the left lung. **(A)** Representative images of left lung tumors formed after injecting 100 flow sorted B931 SP CSCs with 500 CAFs or NAFs. Mice were treated with vehicle or TGFβi as indicated. **(B)** Quantification of left lung tumor volumes at the time of harvest. ^*ns*^*P* > 0.05, ***P < 0.001.

Since CAFs aid SCC expansion without themselves expanding, we next assessed if SCC cell expansion promoted by CAFs in the lung is due to CAFs’ effects on CSC self-renewal and survival *in vivo*. We sorted the SP cells to define CSCs, a subpopulation of metastasis-associated CSCs, as previously described (White et al., 2013), and co-injected 100 SP cells with 5,000 CAFs or NAFs to the left lung. CAFs, but not NAFs, increased tumor volumes in either C57BL/6J or athymic recipients (Figures 4D–G), suggesting that CAFs’ effect on lung CSC expansion does not require T-cells. Fluorescent dissecting microscopy confirmed that the gross volumes were primarily GFP^+^ SCC cells (Figure 4E), suggesting that CAFs do not themselves expand but facilitate CSC cell expansion in the lung. Treating mice with the TGFβ inhibitor LY2157299 reduced lung SCC volumes in CSCs co-injected with CAFs (Figure 5), suggesting that TGFβ signaling is critical to CAFs influence on CSC survival and/or expansion in the lung.

### CAF-Produced TGFβ Promotes CSC Self-Renewal and Invasion

**FIGURE 6:**
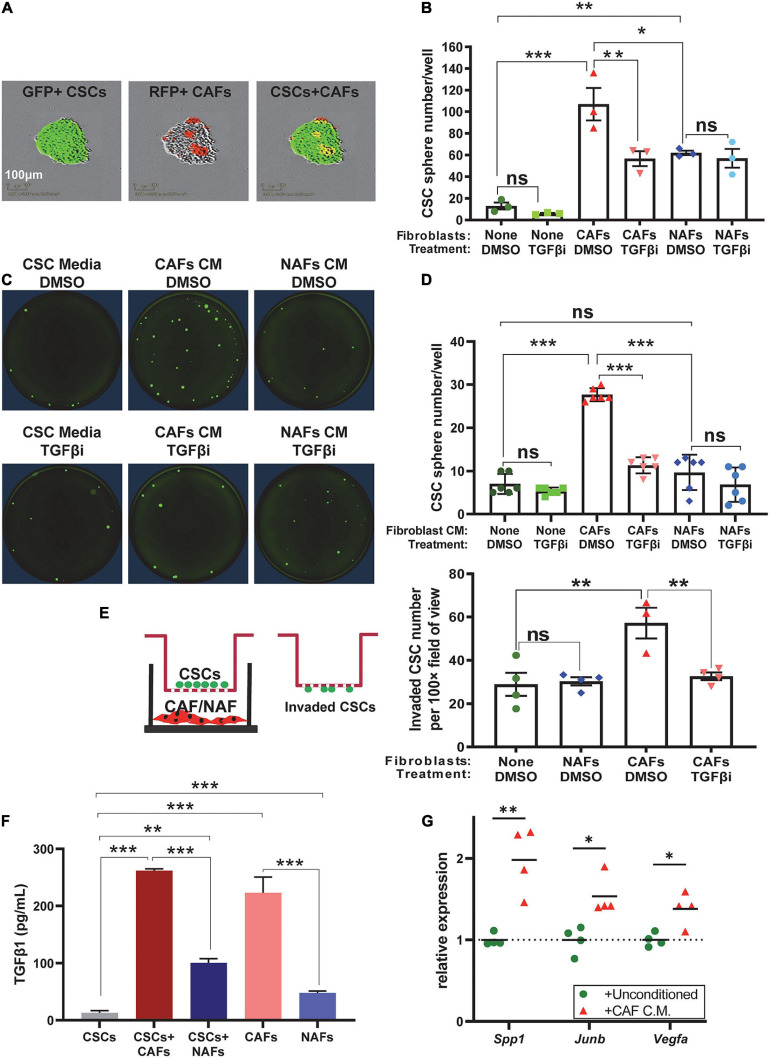
Cancer-associated fibroblasts increased self-renewal and invasion of CSCs in a TGFβ-dependent manner *via* direct contact and paracrine effects. **(A)** Representative images of GFP^+^ A223 CSCs and RFP^+^ CAFs in spheroid co-culture. **(B)** Quantification of sphere number in each of the indicated A223 CSC treatment [vehicle (DMSO) or TGFβ inhibitor (“TGFβi”)] ± fibroblast co-culture conditions. **(C,D)** Representative images and quantification of A223 CSC spheres cultured with the indicated conditioned media (CM) and vehicle (DMSO) or TGFβi. **(E)** Transwell invasion assay: A223 CSCs were seeded in the top Matrigel-coated chamber, and CAFs or NAFs were cultured in the bottom well. After 48 h, invaded CSCs attached to the underside of the upper chamber were quantified. **(F)** TGFβ1 concentration in each of the indicated CM was determined by ELISA. Either three or four technical replicates were conducted for each cell type. **(G)** The CM of CAFs or unconditioned control media was applied to recipient A223 cells in a 24 h sphere initiation assay. RNA harvested from A223 recipient cells was evaluated by RT-qPCR for the expression of TGFβ-target genes *Spp1*, *Junb*, and *Vegfa* normalized to the expression of *Gapdh*. ^*ns*^*P* > 0.05, *P < 0.05, **P < 0.01, ***P < 0.001.

To determine if CAFs directly affect CSC self-renewal, we performed sphere formation assays in ULA plates. A total of 100 GFP^+^ SP cells/well were co-cultured with 500 RFP^+^ CAFs or NAFs per well in six-well plates. Because CAFs or NAFs were integrated into the spheres, only spheres with >50 GFP^+^ CSCs/sphere were counted 7–10 days after culture. Both NAFs and CAFs increased the abundance of spheres, but CAF co-cultures had higher sphere numbers than NAF co-cultures (Figures 6A,B). Adding TGFβ inhibitor LY2157299 to the culture media (5 μmol/L) attenuated sphere formation in CSC-CAF co-cultures, but not CSC alone or CSC-NAF co-culture (Figure 6B), suggesting that TGFβ supplied by CAFs is responsible for increased sphere formation induced by CAFs. To assess if cell–cell contact is required for CAFs to enhance CSC sphere formation, we measured CSC sphere formation as a function of CM from CAFs or NAFs. CM from CAFs but not NAFs significantly increased sphere formation that was attenuated by TGFβ inhibitor (Figures 6C,D).

Because we previously demonstrated increased metastasis in mice with higher numbers of SP CSCs (White et al., 2013), we assessed if CAFs affect CSC invasion using transwell invasion chambers. We plated SP cells in the top chamber of Matrigel-coated membranes, and plated CAFs or NAFs in the bottom chamber, and quantified CSC invasion to the underside of the membrane. CAFs, but not NAFs enhanced CSC invasion and the effect was attenuated by TGFβ inhibition (Figure 6E).

Since CAFs were not in direct contact with CSCs (Figures 6C–E), spheres represent SCC cells alone and factors released from CAFs must have contributed to CSC sphere formation and invasion. Indeed, TGFβ1 ELISA showed that CAFs are the primary source of secreted TGFβ1 in conditioned culture media (Figure 6F). Finally, to assess if CAFs drive TGFβ signaling in neighboring SCC cells, we applied the CM from CAFs to recipient SCC cells in a short-term sphere culture and found that CAF CM increased the expression of know TGFβ target genes, *Spp1*, *Junb*, and *Vegfa* (Figure 6G). These TGFβ target gene products are involved in self-renewal, metastasis, clonal expansion, and angiogenesis (Gokulnath et al., 2017; Kim et al., 2017; Sui et al., 2017; Huang et al., 2019; Kallergi et al., 2019).

### TGFβ Activation and CAFs Correlates With Metastasis in Human Oral SCCs and Mice

**FIGURE 7:**
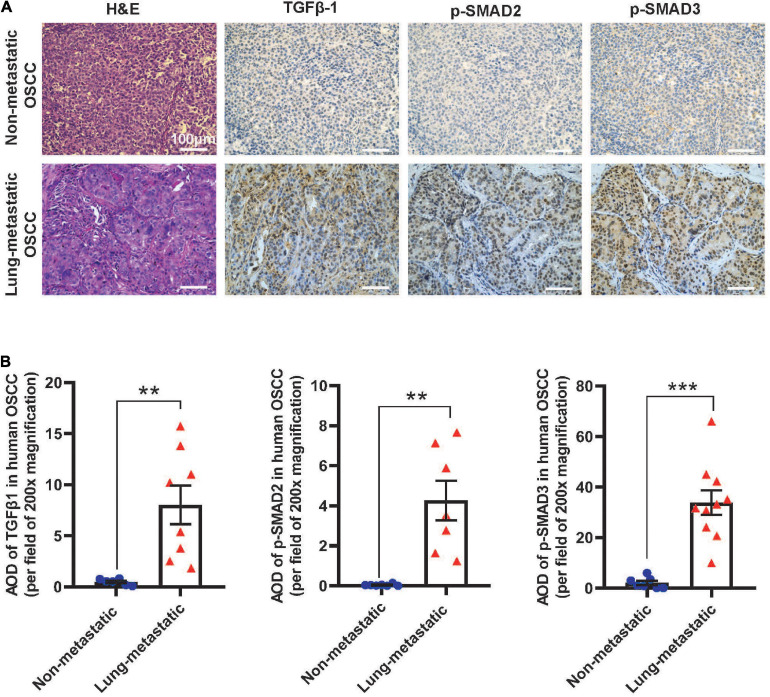
TGFβ signaling is activated in primary human OSCC tumors in patients with lung metastasis. **(A)** Representative H&E and IHC images examining expression of the indicated markers in human OSCC. Upper panels are staining of the primary tumor of a representative patient without metastasis and lower panels are staining of the primary tumor from a representative patient with metastasis. Scale bar: 100 μm. **(B)** Quantification of TGFβ1, p-SMAD2, and p-SMAD3 staining by AOD value. **P < 0.01, ***P < 0.001.

**FIGURE 8:**
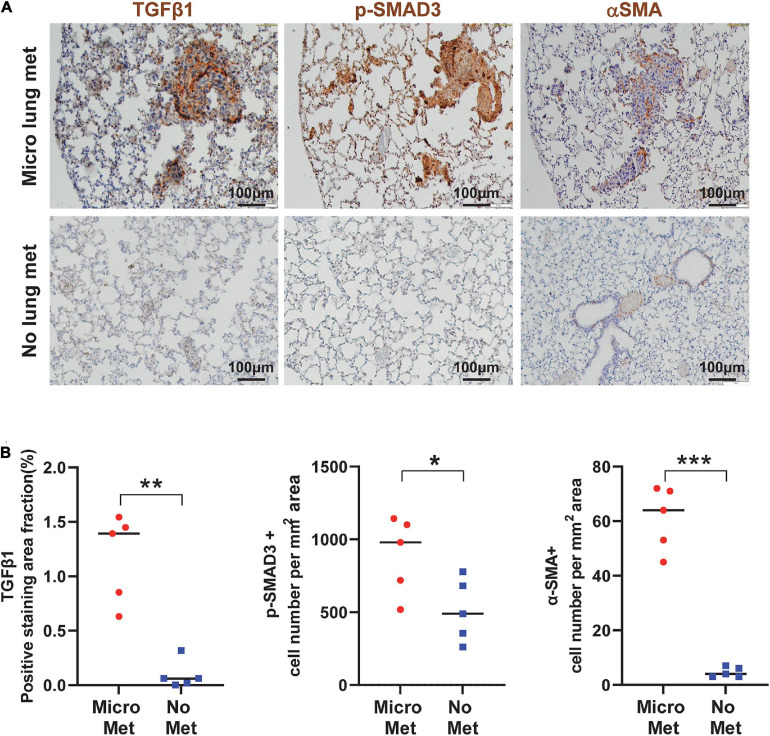
TGFβ signaling is activated in the primary tumor associated with metastasis and the metastatic niche. **(A)** Representative IHC staining of TGFβ1, p-SMAD3, and αSMA in lungs from mice with or without micrometastasis at 4–5 weeks after B931 cell flank transplantation. Scale bar: 100 μm. **(B)** Quantification of TGFβ1, p-SMAD3, and αSMA in lungs with or without B931 micrometastasis. *P < 0.05, **P < 0.01, ***P < 0.001.

To assess if our findings in mouse models apply to human SCCs, we performed IHC staining of TGFβ1, p-SMAD2, and p-SMAD3 in primary tumor oral SCC clinical specimens from patients with or without lung metastasis. The staining intensity of these proteins in primary SCCs in patients with metastasis were significantly higher than SCCs in patients without metastasis (Figures 7A,B), supporting the notion that TGFβ activation in primary SCC cells might be a critical aspect of metastasis. Additionally, in mouse lungs with SCC micrometastasis from implanted flank SCCs, activated αSMA+ fibroblasts were coincidental with strong TGFβ1 and p-SMAD3, which were not evident in lungs without micrometastasis in tumor-bearing mice (Figures 8A,B), further suggesting the critical role of TGFβ1 in activating fibroblasts to prepare the metastatic niche.

## Discussion

### CAFs Co-transplanted With SCCs Did Not Promote Primary SCC Growth or Trafficking but Enhanced SCC Lung Seeding and Expansion

In this study, we assessed the effect of CAFs on SCC formation and metastasis. While CAFs are reported to promote SCC growth (Orimo et al., 2005; Erez et al., 2010), the primary SCC growth in our model of transplantation of bulk SCC cells was not affected by CAFs. It is possible that SCCs in our model are aggressive with Kras^G12D^-dependent autonomous growth further sustained by homozygous *Smad4* loss. Because of their aggressive behaviors, these SCCs could either be self-sustained or rapidly educate normal fibroblasts in their natural location to form CAFs in the native microenvironment. Consistent with this, the CAF cell lines used in these studies were generated by harvesting the CAFs from transplanted SCCs, demonstrating the ability of the tumor to generate CAFs from the normal mouse microenvironment. Further, CAFs did not appear to protect and aid SCC cell trafficking and extravasation as i.v. co-injection of SCCs/CAFs did not significantly increase the number of lung nodules seeded by tumor cells (Supplementary Figure 2). Given these results, it is likely that SCC cells themselves have already acquired survival ability prior to dissemination and trafficking as we previously observed (Wu et al., 2019).

In contrast, CAFs promoted SCC cell expansion in the lung as compared to the same numbers of SCC and CAF cells co-transplanted at primary site. This contribution from CAFs appears to be linked to increased TGFβ1 (Figure 1) that could impact property changes of both SCC cells and CAFs. This notion is supported by data demonstrating that TGFβ inhibitor attenuated CAF’s effects on SCC cell seeding to the lung (Figures 2, 5), a foreign microenvironment for SCC cells to find their niche for establishment. This could explain the difference between no obvious effects of CAFs at the primary site but profound effects at the metastatic site. αSMA+ fibroblasts’ presence in the lung-transplanted tumors (Figure 2C) further consolidated our notion that CAFs were a source of TGFβ1. It seemed that only co-injected CAFs instead of tumor cells alone can efficiently integrate more newly generated CAFs in the tumor, because only lung tumors derived from SCC/CAF co-injection have CAFs in the tumor core (Figure 2C). Since CAFs secreted higher TGFβ1 level than tumor cells or NAFs (Figure 1E) and TGFβ1 increased CAFs’ migration (Karagiannis et al., 2014), we inferred one reason for this was that SCC/CAF secreted higher amount of TGFβ1 which increased fibroblast activation, and this enabled CAFs to establish in the tumor core.

The expansion of SCC volumes in the lung by CAFs could be due to reduced apoptosis, increased angiogenesis, and increased proliferation, all of which appeared to be contributed by TGFβ because TGFβ inhibition attenuated these CAF effects. Our results demonstrating that TGFβ inhibition reduced apoptosis in SCC cells are consistent with those reported in other cancer types. For example, TGFβ1 protects colon cancer cells from apoptosis (Moon et al., 2019) and TGFβ downregulation induced cancer cell apoptosis in melanoma and pancreas adenocarcinoma (Han et al., 2018). Our results suggested that elevated apoptosis could result from reduced angiogenesis, and this was in line with Folkman’s finding (Folkman, 2003). In contrast, *Smad4* mutant SCC cells, including CSCs, have lost responsiveness to TGFβ-induced growth arrest which requires SMAD4 (Wu et al., 2018). This may explain why TGFβ inhibition did not affect SCC proliferation without CAFs. Therefore, the effect of TGFβ inhibition on reducing SCC proliferation induced by CAFs is likely to be mediated by additional growth factors produced in CAFs instead of in SCC cells.

Cancer-associated fibroblasts appeared to primarily affect CSCs, as co-injection of CAFs with CSCs have effects on CSC seeding to the lung similar to (or greater than) unselected SCC cells (Figures 2A,B vs. Figures 4D–G). Further, CAFs’ effects were comparable in immune-compromised vs. competent background (Figures 4D–G). It is possible that these SCC cells in the lung can rapidly induce immune suppression in a fashion similar to the primary tumor site as we previously observed (Mishra et al., 2016). Therefore, CAFs’ effects are, to some extent, T cell-independent.

### CAFs Promoted CSC Expansion and Invasion/Migration *via* Direct Contact and Paracrine Effects

The large absence of labeled CAFs in SCC lesions in the lung (Figures 4A,H) suggests that CAFs primarily provide a physical niche for CSCs and that CAFs do not proliferate with the tumor cells. This is evidenced by CSC self-renewal primarily expanding tumor cells with much fewer numbers of CAFs in CSC spheres (Figure 6A). Additionally, CAFs provide paracrine effects on promoting CSC expansion, evidenced by increased CSC spheres when CSCs were exposed to CAF conditioned culture media. The effects appear dependent upon TGFβ ligand, as TGFβ inhibitor attenuated this CAF-mediated CSC expansion. Our previous data have shown data that SP cells, but not CD49f+ CSCs, are a subpopulation of metastasis associated CSCs (White et al., 2013). Hence, we used sorted SP cells directly, but not CD49f+ CSCs, and our data provide mechanistic and *in vivo* validation that TGFβ signaling similarly promotes self-renewal of *Smad4* mutant SCC and does so *via* CAFs. CAFs also promoted TGFβ-associated CSC migration/invasion (Figure 6E), which could contribute to the increased sizes of lung SCC lesions by CAFs *in vivo* (Figures 2, 4, 5). In this specific model, TGFβ produced from CAFs can exert paracrine effects on CSCs *via* SMAD2/3-dependent, SMAD4-independent mechanisms (Oshimori et al., 2015; Yang et al., 2020), or non-canonical TGFβ signaling (Li et al., 2019; Woosley et al., 2019). Our data are consistent with previous reports that in several other cancer types, TGFβ activation promotes CSC properties including self-renewal (Woosley et al., 2019) and invasiveness (Oshimori et al., 2015). TGFβ responding-CSCs are apoptosis-resistant (Oshimori et al., 2015) and induce the exhaustion of cytotoxic T cells (Miao et al., 2019), all of which benefit CSC expansion in the lung.

### CAFs Could Be a Major Target of TGFβ Inhibition for Treating Advanced SCC

TGFβ1 is often overexpressed in cancer (Calon et al., 2012; Pickup et al., 2013; Wu et al., 2020; Yegodayev et al., 2020). Our current study demonstrated that CAFs could produce TGFβ1 at higher levels than tumor epithelial cells (Figures 1E, 6F), and TGFβ signaling plays a major role in CSC expansion in a foreign (metastatic) microenvironment. Although these SCC models induce CAF formation in the primary tumor and we cannot discern the actions of co-transplanted CAFs vs. the actions of newly formed CAFs in the primary tumor or the metastatic microenvironment, the direct conditioning of the lung microenvironment by co-transplanted CAFs to increase lung colonization in a TGFβ-dependent manner suggests that CAF-induced TGFβ signaling is a critical step. The correlation between TGFβ pathway activation and metastasis status of human SCCs in this study further validates the translatability of our mouse SCC models, suggesting that targeting TGFβ signaling, even in severely immune-compromised SCCs and *Smad4* mutant SCC, could have two benefits: (1) inhibition of CSC properties; (2) inhibition of invasion/metastasis/niche preparation. These notions are further supported by previous findings that primary SCCs with metastasis possess more activated fibroblasts and CAFs than those without metastasis, and that TGFβ activation increases activated fibroblast/CAF numbers that in turn secrete more TGFβ1 (Sridhara et al., 2013; Luksic et al., 2015; Maqsood et al., 2020). Because biopsy of SCC lung metastasis in patients is generally not feasible, it is difficult to draw a definitive conclusion using human specimens whether TGFβ1 is sufficient to generate a paracrine/systemic effect for metastatic niche preparation, but our mouse model demonstrates a correlation between TGFβ ligands and micrometastasis. Since i.v. CSCs/CAFs co-injection in our mouse model did not increase CSC seeding to the lung, it suggests that systemic TGFβ1 secreted from primary SCCs (including their resident CAFs) could facilitate CAF formation in the lung either at the pre-metastatic niche or metastatic niche after SCC are seeded. Future studies will identify molecular and cellular mechanisms of metastatic or pre-metastatic niche development contributed by CAFs and TGFβ.

In summary, our study identified that CAFs promote CSC properties for them to survive and expand at the foreign, metastatic microenvironment. TGFβ1 ligand produced by CAFs is the dominant driving force of these effects. Our findings compel future studies examining the premetastatic and metastatic niche promoted by CAFs so that treating SCC metastasis in patients can be explored.

## Data Availability Statement

The datasets presented in this study can be found in online repositories. The names of the repository/repositories and accession numbers can be found below: https://www.ncbi.nlm.nih.gov/biosample/18349411; https://www.ncbi.nlm.nih.gov/biosample/18349412; and https://www.ncbi.nlm.nih.gov/sra/PRJNA715402.

## Ethics Statement

The animal study was reviewed and approved by Institutional Animal Care and Use Committee of the University of Colorado Anschutz Medical Campus.

## Author Contributions

XS, JL, and CY performed experiments, analyzed data, and generated figures. DD, FW, SH, and KW performed experiments. MR performed data analysis. X-JW, CY, and HZ designed and supervised the study. XS, JL, HZ, CY, and X-JW wrote the manuscript. All authors contributed to the article and approved the submitted version.

## Conflict of Interest

The authors declare that the research was conducted in the absence of any commercial or financial relationships that could be construed as a potential conflict of interest.

## Publisher’s Note

All claims expressed in this article are solely those of the authors and do not necessarily represent those of their affiliated organizations, or those of the publisher, the editors and the reviewers. Any product that may be evaluated in this article, or claim that may be made by its manufacturer, is not guaranteed or endorsed by the publisher.

## Footnotes

1
https://www.ncbi.nlm.nih.gov/sra

## References

[B1] Alfieri S. Carenzo A. Platini F. Serafini M. S. Perrone F. Galbiati D. (2020). Tumor biomarkers for the prediction of distant metastasis in head and neck squamous cell carcinoma. *Cancers* 12:922. 10.3390/cancers12040922 32283719 PMC7225924

[B2] Álvarez-Teijeiro S. Garcia-Inclan C. Villaronga M. A. Casado P. Hermida-Prado F. Granda-Duaz R. (2018). Factors secreted by cancer-associated fibroblasts that sustain cancer stem properties in head and neck squamous carcinoma cells as potential therapeutic targets. *Cancers* 10:334. 10.3390/cancers10090334 30227608 PMC6162704

[B3] Bohnenberger H. Kaderali L. Ströbel P. Yepes D. Plessmann U. Dharia N. V. (2018). Comparative proteomics reveals a diagnostic signature for pulmonary head-and-neck cancer metastasis. *EMBO Mol. Med.* 10:e8428. 10.15252/emmm.201708428 30097507 PMC6127892

[B4] Calon A. Espinet E. Palomo-Ponce S. Tauriello D. V. F. Iglesias M. Céspedes M. V. (2012). Dependency of colorectal cancer on a TGF-β-driven program in stromal cells for metastasis initiation. *Cancer Cell* 22 571–584. 10.1016/j.ccr.2012.08.013 23153532 PMC3512565

[B5] Chaffer C. L. Weinberg R. A. (2011). A perspective on cancer cell metastasis. *Science* 331 1559–1564. 10.1126/science.1203543 21436443

[B6] Chen W.-J. Ho C.-C. Chang Y.-L. Chen H.-Y. Lin C.-A. Ling T.-Y. (2014). Cancer-associated fibroblasts regulate the plasticity of lung cancer stemness via paracrine signalling. *Nat. Commun.* 5:3472. 10.1038/ncomms4472 24668028

[B7] Erez N. Truitt M. Olson P. Arron S. T. Hanahan D. (2010). Cancer-associated fibroblasts are activated in incipient neoplasia to orchestrate tumor-promoting inflammation in an NF-κB-dependent manner. *Cancer Cell* 17 135–147. 10.1016/j.ccr.2009.12.041 20138012

[B8] Folkman J. (2003). Angiogenesis and apoptosis. *Semin. Cancer Biol.* 13 159–167. 10.1016/s1044-579x(02)00133-5 12654259

[B9] Gokulnath M. Swetha R. Thejaswini G. Shilpa P. Selvamurugan N. (2017). Transforming growth factor-β1 regulation of ATF-3, c-Jun and JunB proteins for activation of matrix metalloproteinase-13 gene in human breast cancer cells. *Int. J. Biol. Macromol.* 94 370–377. 10.1016/j.ijbiomac.2016.10.026 27751807

[B10] Han Z. Kang D. Joo Y. Lee J. Oh G.-H. Choi S. (2018). TGF-β downregulation-induced cancer cell death is finely regulated by the SAPK signaling cascade. *Exp. Mol. Med.* 50 1–9. 10.1038/s12276-018-0189-8 30523245 PMC6283885

[B11] Hogervorst M. Rietveld M. De Gruijl F. El Ghalbzouri A. (2018). A shift from papillary to reticular fibroblasts enables tumour-stroma interaction and invasion. *Br. J. Cancer* 118 1089–1097. 10.1038/s41416-018-0024-y 29551776 PMC5931114

[B12] Hu H. Tian D. Chen T. Han R. Sun Y. Wu C. (2014). Metastasis-associated in colon cancer 1 is a novel survival-related biomarker for human patients with renal pelvis carcinoma. *PLoS One* 9:e100161. 10.1371/journal.pone.0100161 24949951 PMC4064998

[B13] Huang J. Chang S. Lu Y. Wang J. Si Y. Zhang L. (2019). Enhanced osteopontin splicing regulated by RUNX2 is HDAC-dependent and induces invasive phenotypes in NSCLC cells. *Cancer Cell Int.* 19:306. 10.1186/s12935-019-1033-5 31832019 PMC6873507

[B14] Kallergi G. Tsintari V. Sfakianakis S. Bei E. Lagoudaki E. Koutsopoulos A. (2019). The prognostic value of JUNB-positive CTCs in metastatic breast cancer: from bioinformatics to phenotypic characterization. *Breast Cancer Res.* 21:86. 10.1186/s13058-019-1166-4 31370904 PMC6676640

[B15] Kalluri R. (2016). The biology and function of fibroblasts in cancer. *Nat. Rev. Cancer* 16 582–598. 10.1038/nrc.2016.73 27550820

[B16] Karagiannis G. S. Schaeffer D. F. Cho C. K. Musrap N. Saraon P. Batruch I. (2014). Collective migration of cancer-associated fibroblasts is enhanced by overexpression of tight junction-associated proteins claudin-11 and occludin. *Mol. Oncol.* 8 178–195. 10.1016/j.molonc.2013.10.008 24268521 PMC5528551

[B17] Kim M. Jang K. Miller P. Picon-Ruiz M. Yeasky T. M. El-Ashry D. (2017). VEGFA links self-renewal and metastasis by inducing Sox2 to repress miR-452, driving Slug. *Oncogene* 36 5199–5211. 10.1038/onc.2017.4 28504716 PMC5596211

[B18] Le P. N. Keysar S. B. Miller B. Eagles J. R. Chimed T.-S. Reisinger J. (2019). Wnt signaling dynamics in head and neck squamous cell cancer tumor-stroma interactions. *Mol. Carcinog.* 58 398–410. 10.1002/mc.22937 30378175 PMC6460915

[B19] Li A. G. Wang D. Feng X. H. Wang X. J. (2004). Latent TGFbeta1 overexpression in keratinocytes results in a severe psoriasis-like skin disorder. *EMBO J.* 23 1770–1781. 10.1038/sj.emboj.7600183 15057277 PMC394237

[B20] Li H. Zhang J. Chen S.-W. Liu L.-L. Li L. Gao F. (2015). Cancer-associated fibroblasts provide a suitable microenvironment for tumor development and progression in oral tongue squamous cancer. *J. Transl. Med.* 13:198. 10.1186/s12967-015-0551-8 26094024 PMC4475624

[B21] Li K. Yang L. Li J. Guan C. Zhang S. Lao X. (2019). TGFβ induces stemness through non-canonical AKT-FOXO3a axis in oral squamous cell carcinoma. *EBioMedicine* 48 70–80. 10.1016/j.ebiom.2019.09.027 31629677 PMC6838363

[B22] Lu S. L. Herrington H. Reh D. Weber S. Bornstein S. Wang D. (2006). Loss of transforming growth factor-beta type II receptor promotes metastatic head-and-neck squamous cell carcinoma. *Genes. Dev.* 20 1331–1342. 10.1101/gad.1413306 16702406 PMC1472907

[B23] Luksic I. Suton P. Manojlovic S. Virag M. Petrovecki M. Macan D. (2015). Significance of myofibroblast appearance in squamous cell carcinoma of the oral cavity on the occurrence of occult regional metastases, distant metastases, and survival. *Int. J. Oral Maxillofac. Surg.* 44 1075–1080. 10.1016/j.ijom.2015.05.009 26055525

[B24] Luo J. Bian L. Blevins M. A. Wang D. Liang C. Du D. (2019). Smad7 promotes healing of radiotherapy-induced oral mucositis without compromising oral cancer therapy in a xenograft mouse model. *Clin. Cancer Res.* 25 808–818. 10.1158/1078-0432.CCR-18-1081 30185419 PMC6335168

[B25] Malanchi I. Santamaria-Martínez A. Susanto E. Peng H. Lehr H.-A. Delaloye J.-F. (2011). Interactions between cancer stem cells and their niche govern metastatic colonization. *Nature* 481 85–89. 10.1038/nature10694 22158103

[B26] Maqsood A. Ali A. Zaffar Z. Mokeem S. Mokeem S. S. Ahmed N. (2020). Expression of CD34 and α-SMA markers in oral squamous cell carcinoma differentiation. a histological and histo-chemical study. *Int. J. Environ. Res. Public Health* 18:192. 10.3390/ijerph18010192 33383808 PMC7795485

[B27] Markwell S. M. Weed S. A. (2015). Tumor and stromal-based contributions to head and neck squamous cell carcinoma invasion. *Cancers* 7 382–406. 10.3390/cancers7010382 25734659 PMC4381264

[B28] Martin C. J. Datta A. Littlefield C. Kalra A. Chapron C. Wawersik S. (2020). Selective inhibition of TGFβ1 activation overcomes primary resistance to checkpoint blockade therapy by altering tumor immune landscape. *Sci. Transl. Med.* 12:8456., 10.1126/scitranslmed.aay8456 32213632

[B29] Mazzocca A. Fransvea E. Dituri F. Lupo L. Antonaci S. Giannelli G. (2010). Down-regulation of connective tissue growth factor by inhibition of transforming growth factor beta blocks the tumor-stroma cross-talk and tumor progression in hepatocellular carcinoma. *Hepatology* 51 523–534. 10.1002/hep.23285 19821534

[B30] Meng W. Wu Y. He X. Liu C. Gao Q. Ge L. (2014). A systems biology approach identifies effective tumor-stroma common targets for oral squamous cell carcinoma. *Cancer Res.* 74 2306–2315. 10.1158/0008-5472.CAN-13-2275 24556718

[B31] Miao Y. Yang H. Levorse J. Yuan S. Polak L. Sribour M. (2019). Adaptive immune resistance emerges from tumor-initiating stem cells. *Cell* 177:25. 10.1016/j.cell.2019.03.025 31031009 PMC6525024

[B32] Mishra A. K. Kadoishi T. Wang X. Driver E. Chen Z. Wang X.-J. (2016). Squamous cell carcinomas escape immune surveillance via inducing chronic activation and exhaustion of CD8+ T Cells co-expressing PD-1 and LAG-3 inhibitory receptors. *Oncotarget* 7 81341–81356. 10.18632/oncotarget.13228 27835902 PMC5340255

[B33] Moon J. R. Oh S. J. Lee C. K. Chi S. G. Kim H. J. (2019). TGF-β1 protects colon tumor cells from apoptosis through XAF1 suppression. *Int. J. Oncol.* 54 2117–2126. 10.3892/ijo.2019.4776 31081052

[B34] Orimo A. Gupta P. B. Sgroi D. C. Arenzana-Seisdedos F. Delaunay T. Naeem R. (2005). Stromal fibroblasts present in invasive human breast carcinomas promote tumor growth and angiogenesis through elevated SDF-1/CXCL12 secretion. *Cell* 121 335–348. 10.1016/j.cell.2005.02.034 15882617

[B35] Oshimori N. Oristian D. Fuchs E. (2015). TGF-β promotes heterogeneity and drug resistance in squamous cell carcinoma. *Cell* 160 963–976. 10.1016/j.cell.2015.01.043 25723170 PMC4509607

[B36] Peltanova B. Raudenska M. Masarik M. (2019). Effect of tumor microenvironment on pathogenesis of the head and neck squamous cell carcinoma: a systematic review. *Mol. Cancer* 18:63. 10.1186/s12943-019-0983-5 30927923 PMC6441173

[B37] Pickup M. Novitskiy S. Moses H. L. (2013). The roles of TGFβ in the tumour microenvironment. *Nat. Rev. Cancer* 13 788–799. 10.1038/nrc3603 24132110 PMC4025940

[B38] Plaks V. Kong N. Werb Z. (2015). The cancer stem cell niche: how essential is the niche in regulating stemness of tumor cells? *Cell Stem Cell* 16 225–238. 10.1016/j.stem.2015.02.015 25748930 PMC4355577

[B39] Presby D. M. Checkley L. A. Jackman M. R. Higgins J. A. Jones K. L. Giles E. D. (2019). Regular exercise potentiates energetically expensive hepatic de novo lipogenesis during early weight regain. *Am. J. Physiol. Regul. Integr. Comp. Physiol.* 317 R684–R695. 10.1152/ajpregu.00074.2019 31553623 PMC6879845

[B40] Sridhara S. U. Choudaha N. Kasetty S. Joshi P. S. Kallianpur S. Tijare M. (2013). Stromal myofibroblasts in nonmetastatic and metastatic oral squamous cell carcinoma: An immunohistochemical study. *J. Oral. Maxillofac. Pathol.* 17 190–194. 10.4103/0973-029x.119758 24250077 PMC3830225

[B41] Su S. Chen J. Yao H. Liu J. Yu S. Lao L. (2018). CD10GPR77 cancer-associated fibroblasts promote cancer formation and chemoresistance by sustaining cancer stemness. *Cell* 172:9 10.1016/j.cell.2018.01.009 29395328

[B42] Sui H. Zhao J. Zhou L. Wen H. Deng W. Li C. (2017). Tanshinone IIA inhibits β-catenin/VEGF-mediated angiogenesis by targeting TGF-β1 in normoxic and HIF-1α in hypoxic microenvironments in human colorectal cancer. *Cancer Lett.* 403 86–97. 10.1016/j.canlet.2017.05.013 28602978

[B43] Tran H. C. Wan Z. Sheard M. A. Sun J. Jackson J. R. Malvar J. (2017). TGFβR1 Blockade with Galunisertib (LY2157299) enhances anti-neuroblastoma activity of the Anti-GD2 antibody dinutuximab (ch14.18) with natural killer cells. *Clin. Cancer Res.* 23 804–813. 10.1158/1078-0432.Ccr-16-1743 27756784 PMC5361893

[B44] White R. A. Neiman J. M. Reddi A. Han G. Birlea S. Mitra D. (2013). Epithelial stem cell mutations that promote squamous cell carcinoma metastasis. *J. Clin. Investig.* 123 4390–4404. 10.1172/JCI65856 23999427 PMC3784525

[B45] Woosley A. N. Dalton A. C. Hussey G. S. Howley B. V. Mohanty B. K. Grelet S. (2019). TGFβ promotes breast cancer stem cell self-renewal through an ILEI/LIFR signaling axis. *Oncogene* 38 3794–3811. 10.1038/s41388-019-0703-z 30692635 PMC6525020

[B46] Wu F. Weigel K. J. Zhou H. Wang X. J. (2018). Paradoxical roles of TGF-beta signaling in suppressing and promoting squamous cell carcinoma. *Acta Biochim. Biophys. Sin.* 50 98–105. 10.1093/abbs/gmx127 29206939 PMC5846704

[B47] Wu F. L. Nolan K. Strait A. A. Bian L. Nguyen K. A. Wang J. H. (2019). Macrophages promote growth of squamous cancer independent of T cells. *J. Dent. Res.* 98 896–903. 10.1177/0022034519854734 31189369 PMC6616122

[B48] Wu P. Geng B. Chen Q. Zhao E. Liu J. Sun C. (2020). Tumor cell-derived TGFβ1 attenuates antitumor immune activity of T cells via Regulation of PD-1 mRNA. *Cancer Immunol. Res.* 2020:113. 10.1158/2326-6066.CIR-20-0113 32999004

[B49] Yang L. Shi P. Zhao G. Xu J. Peng W. Zhang J. (2020). Targeting cancer stem cell pathways for cancer therapy. *Signal Transduct. Targeted Ther.* 5:8. 10.1038/s41392-020-0110-5 32296030 PMC7005297

[B50] Yang X. Lin Y. Shi Y. Li B. Liu W. Yin W. (2016). FAP Promotes immunosuppression by cancer-associated fibroblasts in the tumor microenvironment via STAT3-CCL2 Signaling. *Cancer Res.* 76 4124–4135. 10.1158/0008-5472.CAN-15-2973 27216177

[B51] Yegodayev K. M. Novoplansky O. Golden A. Prasad M. Levin L. Jagadeeshan S. (2020). TGF-beta-activated cancer-associated fibroblasts limit cetuximab efficacy in preclinical models of head and neck cancer. *Cancers* 12:339. 10.3390/cancers12020339 32028632 PMC7073231

