# Supplementary material for: Cancer-Associated Fibroblasts Facilitate Squamous Cell Carcinoma Lung Metastasis in Mice by Providing TGFβ-Mediated Cancer Stem Cell Niche
Source: Front Cell Dev Biol. 2021 Aug 30;9:668164. doi: 10.3389/fcell.2021.668164 (PMC8435687; doi:10.3389/fcell.2021.668164)
Supplement: Supplementary file 1 [file Data_Sheet_1.PDF]

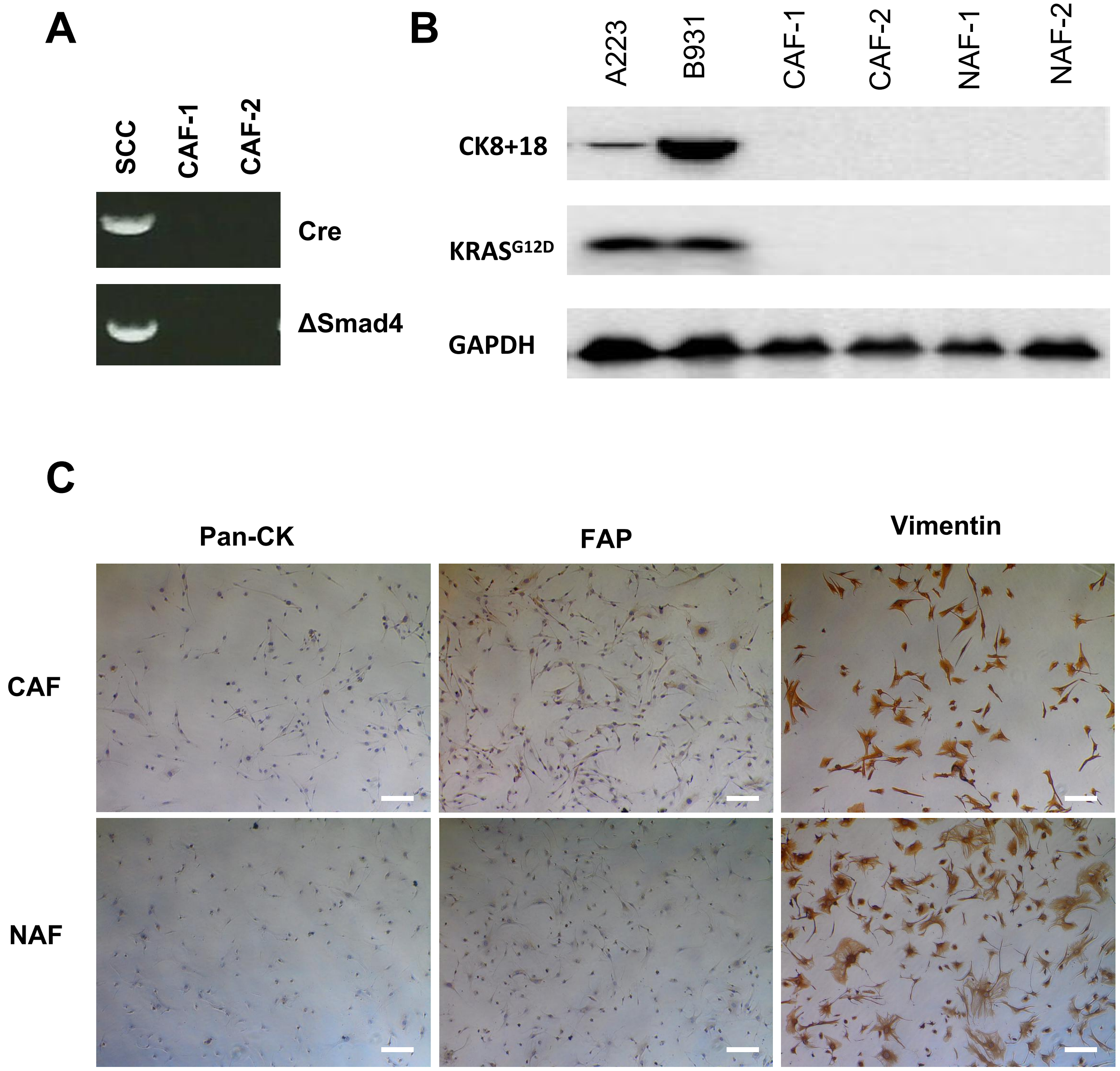

Supplementary Figure 1. CAFs and NAFs were free of contaminated SCC cells or epithelial cells. (A) PCR results for detection of K15.CrePR1 and Smad4 deletion. **(B)** The lysates of A223 or B931 SCC cells or the indicated CAF and NAF cell lines were evaluated by western blot with the indicated antibodies. **(C)** CAF or NAF cell lines were evaluated by immunocytochemistry using the indicated antibodies. Scale bar: 200 μm.

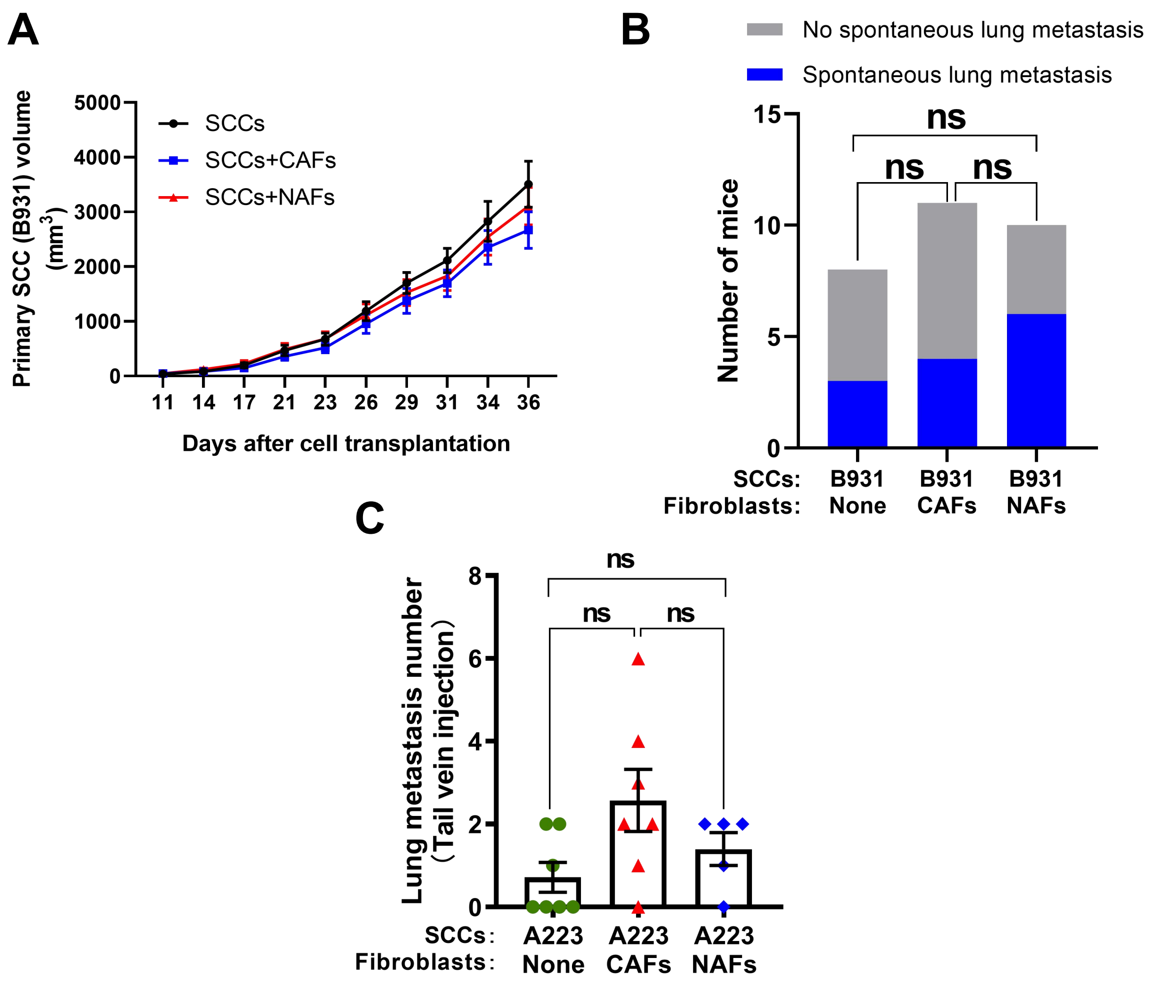

Supplementary Figure 2. CAFs did not influence primary SCC growth or lung metastasis from the primary site or through trafficking. (A) B931 SCC cells with or without CAFs or NAFs were transplanted subcutaneously to the flanks of nude mice. Tumor volume was calculated over time. **(B)** When flank tumors reached about 4000 mm^3 on day 36, mice were sacrificed and their lungs harvested for gross and microscopic metastasis quantification. No difference in spontaneous lung metastasis incidence rates were observed. P values were analyzed by Fisher's exact test. **(C)** A223 SCC cells were co-transplanted with or without CAFs and NAFs via tail vein injection. After 28 days, mice were euthanized and their lungs harvested for determination of metastatic burden. Lung metastasis tumor number quantification was based on gross lung samples.

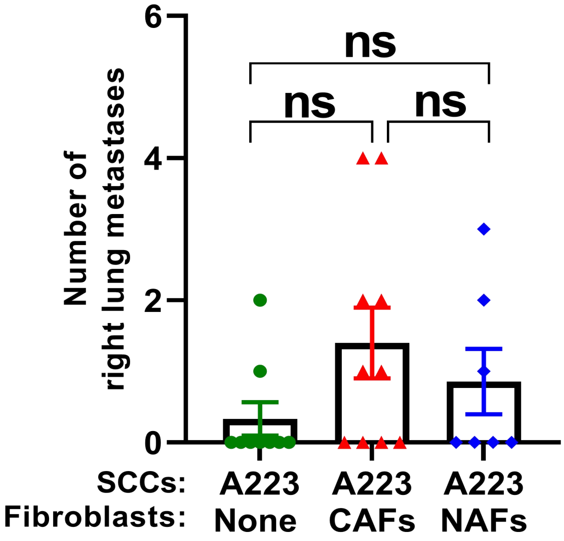

Supplementary Figure 3. CAFs did not affect metastasis to the right lung after cells were directly transplanted in the left lung. A223 SCC cells were transplanted with or without CAFs or NAFs directly to the left lung. Mice were euthanized and lungs harvested on day 21. Quantification of the number of lung metastasis to the right lung was quantified from H&E stained slides.
