# Supplementary material for: Cancer-Associated Fibroblasts Facilitate Squamous Cell Carcinoma Lung Metastasis in Mice by Providing TGFβ-Mediated Cancer Stem Cell Niche
Source: Front Cell Dev Biol. 2021 Aug 30;9:668164. doi: 10.3389/fcell.2021.668164 (PMC8435687; doi:10.3389/fcell.2021.668164)
Supplement: Supplementary file 2 [file Data_Sheet_2.docx]

**Supplementary Materials and Methods**

**Cancer-associated fibroblasts (CAF)/ normal‐associated fibroblasts (NAFs) isolation, culture, purification**

Two thousand A223 cells were suspended in a final volume of 40 μL in 50% Matrigel (Corning)/50% phosphate-buffered saline (PBS) and injected submucosally into the middle part of tongue using a syringe with a 30-gauge needle (Luo et al., 2019). When tumors grew to approximately 150 mm^3^, they were harvested and minced into fine (~1 mm) pieces to isolate CAFs as previously reported (Mazzocca et al., 2010;Yang et al., 2016) and described below. With the same procedures, NAFs were isolated from normal C57BL/6 mouse tongue tissue. Minced tumor/normal tongue pieces were transferred into 50mL conical tubes and incubated at 37°C with 10 mL of DMEM/F12 media containing 0.14 Wunsch units/mL Liberase Blendzyme 3 (Roche) and 1X antibiotic/antimycotic, shaking slowly for 40 minutes. Complete media (15%FBS in DMEM/F12 media with 1×antibiotic/antimycotic) was added to stop Liberase digestion. Cells were filtered through a 70μm filter, the filter washed with media and cells isolated from supernatant by centrifugation (300 xg). Cell pellets were resuspended in complete media and plated in 10 cm culture dishes at 5% CO2, 37°C. To enrich fibroblasts, cell monolayers were washed with PBS and incubated 2 minutes with TrypLE (Gibco). Early detaching, floating fibroblasts were collected while cancer cells remained adherent. This selection was repeated by three to five subsequent subcultures or as needed to remove cancer/epithelial cells contamination.

Spindle shaped fibroblast-like cells were further verified free from K15.*Kras^G12D^*.*Smad4-/-* cancer cells and epithelial cells by PCR, western blot, and immunocytochemistry (ICC) staining (as detailed below).

**PCR/Western blot/Immunocytochemistry staining to verify CAF/NAF purity**

*PCR genotyping*

DNA extraction of A223, CAF, and NAF were performed with DirectPCR Lysis Reagent (Viagen Biotech). PCR master mix was prepared with GoTaq Green Master Mix (Promega). Then K15 F1 (upstream) and Cre R1 (downstream) primers were used for K15 Cre DNA detection. *Smad4/8* (upstream) and *Smad8/10* (downstream) primers were used for detecting *Smad4* deletion as previously described (Garcia-Carracedo et al., 2015). The samples were loaded onto agarose gel and electrophoresed in TBE buffer at 100 V for 1 hour. Then, the gels were stained with ethidium bromide and examined using ChemiDoc™ Imaging System (Bio-Rad).

*Western blotting*

CAF/NAF cell lysates were prepared with RIPA lysis buffer (CST) containing Mini EDTA-free protease inhibitor Cocktail (Roche). Protein concentration was measured by Pierce™ BCA Protein Assay Kit (Thermo Scientific). Protein was loaded on NuPAGE™ 4 to 12% protein gels (Invitrogen) and standard blotting was performed. Membranes were incubated with primary antibodies against KRAS^G12D^ (1:1000, CST), Cytokeratin 8+18 (1:1000, Thermofisher,) and GAPDH (1:1000; Cell Signaling Technology) overnight at 4 degrees. HRP–conjugated secondary antibody (1:5000, Cell Signaling Technology) were used. Proteins were visualized after incubation with chemiluminescent substrate (Thermo Fisher) using a ChemiDoc™ Imaging System (Bio-Rad).

*ICC staining*

Cells were fixed with 4% paraformaldehyde (15 minutes at room temperature), permeabilized with 1X PBS with 0.1% Triton X-100 (5 minutes at room temperature) followed by blocking with 10% goat normal serum (1 hour at room temperature), and incubated with Vimentin (1:200, Abcam), α-SMA (1:200, Abcam), FAP-α (1:200, Abcam) , FSP-1 (1:200, Abcam) and pan-Cytokeratin (Pan-CK, Abcam) primary antibody (4℃, overnight) and then with HRP–conjugated secondary antibody (1 hour at room temperature). DAB chromogen was used for visualization of target protein and hematoxylin applied for counterstaining.

**Tumor transplantation and treatment**

*SCC or CSC cell-fibroblasts lung injection*

For SCC cell and fibroblast co-transplantation directly to the lung, mice were anesthetized with isoflurane using a rodent anesthesia machine. The fur was shaved and the skin was disinfected with chlorhexidine gluconate swabs. A 5 mm transverse incision perpendicular to the left lateral axillary line at the level of the xiphoid process was made by using a sterile scalpel and subcutaneous fat tissue was dissected away until the left lung was visualized (Sakamoto et al., 2015). A total of 1,000 total SCC cells or 100 SP cells with or without 5,000 CAFs/NAFs were suspended in a 40 μL solution consisting of 85% PBS/15% Matrigel(corning). The cell suspension was injected into the mouse left lung using a 30-gauge needle with 3-5 mm insertion depth. The skin incision was closed with vetbond tissue adhesive (Fisher). Mice were treated with TGFβ inhibitor (LY2109761, 75 mg/kg/day or LY2157299, 150 mg/kg/day) by oral gavage or an equal volume of vehicle (250 mL sterile distilled water with 2.5 g carboxymethylcellulose, 1.25 g sodium dodecyl sulfate, and 0.213 g povidone) daily for 3-4 wks before being sacrificed. The first treatment occurred immediately after surgery when mice were fully recovered. Lungs were harvested after mice were sacrificed. The lung tumor number was counted and volume was calculated using the following formula: Volume (mm^3^) = (Length×width×thickness)/2.

*SCC cell-fibroblasts tail vein co-injection*

Mice were placed into a plastic restrainer and warmed with a heat lamp to allow for venous dilation. Immediately after venous expansion, a total of 1000 SCC (A223) cells with or without 5,000 CAFs/NAFs suspended in 150 μL of PBS were injected via the lateral tail vein. Immediately after transplant (less than 30 minutes), mice were treated with 150 mg/kg TGFβ inhibitor (LY2109761) or equal volume of vehicle daily for 4 wks before being sacrificed and lungs were harvested. Lung tumor number and volume were counted and measured as previously described.

*SCC cell-fibroblast* subcutaneous *co-transplantation*

A total of 1,000 total SCC (A223 or B931) cells with or without 5,000 CAFs/NAFs were suspended in 40 μL solution consisting of 50% PBS /50% Matrigel (corning). The cell suspension was injected subcutaneously at the right flank after mice were anesthetized. Tumors were measured by caliper twice a week. When mouse tumor reached 2.0 cm in diameter or mouse experienced 15% weight loss within 2 days, the mouse was euthanized to harvest tumors and lungs.

**Supplementary References**

Garcia-Carracedo, D., Yu, C.-C., Akhavan, N., Fine, S.A., Schönleben, F., Maehara, N., et al. (2015). Smad4 loss synergizes with TGFα overexpression in promoting pancreatic metaplasia, PanIN development, and fibrosis. *PloS one* 10**,** e0120851 doi: 10.1371/journal.pone.0120851.

Luo, J., Bian, L., Blevins, M.A., Wang, D., Liang, C., Du, D., et al. (2019). Smad7 Promotes Healing of Radiotherapy-Induced Oral Mucositis without Compromising Oral Cancer Therapy in a Xenograft Mouse Model. *Clinical cancer research : an official journal of the American Association for Cancer Research* 25**,** 808-818 doi: 10.1158/1078-0432.CCR-18-1081.

Mazzocca, A., Fransvea, E., Dituri, F., Lupo, L., Antonaci, S., and Giannelli, G. (2010). Down-regulation of connective tissue growth factor by inhibition of transforming growth factor beta blocks the tumor-stroma cross-talk and tumor progression in hepatocellular carcinoma. *Hepatology (Baltimore, Md.)* 51**,** 523-534 doi: 10.1002/hep.23285.

Sakamoto, S., Inoue, H., Ohba, S., Kohda, Y., Usami, I., Masuda, T., et al. (2015). New metastatic model of human small-cell lung cancer by orthotopic transplantation in mice. *Cancer Sci* 106**,** 367-374 doi: 10.1111/cas.12624.

Yang, X., Lin, Y., Shi, Y., Li, B., Liu, W., Yin, W., et al. (2016). FAP Promotes Immunosuppression by Cancer-Associated Fibroblasts in the Tumor Microenvironment via STAT3-CCL2 Signaling. *Cancer research* 76**,** 4124-4135 doi: 10.1158/0008-5472.CAN-15-2973.
